# A novel combined laparoscopic-endoscopic overstitch technique for pediatric gastric ulcer perforation: an innovative approach to repair of hollow viscus perforations

**DOI:** 10.1093/jscr/rjaf584

**Published:** 2025-08-06

**Authors:** Andrew Huang-Pacheco, Patrick Thomas, Abdalla Zarroug, Adil A Shah

**Affiliations:** Division of Gastroenterology, Department of Pediatrics, University of Nebraska Medical Center and College of Medicine, Omaha, NE, United States; Division of Pediatric Surgery, Department of Surgery, University of Nebraska Medical Center and College of Medicine, Omaha, NE, United States; Division of Pediatric Surgery, Department of Surgery, University of Nebraska Medical Center and College of Medicine, Omaha, NE, United States; Division of Pediatric Surgery, Department of Surgery, University of Nebraska Medical Center and College of Medicine, Omaha, NE, United States; Division of Pediatric Surgery, Department of Surgery, University of Nebraska Medical Center and College of Medicine, Omaha, NE, United States

**Keywords:** advanced endoscopy, overstitch, perforated ulcer, laparoscopy, minimally invasive surgery, pediatric endoscopy, hybrid procedure

## Abstract

We report the first pediatric use of a laparoscopic-assisted endoscopic overstitch technique for managing an acutely perforated gastric ulcer in a 16-year-old female. The patient presented with symptoms consistent with gastrointestinal perforation, confirmed by radiologic pneumoperitoneum. Diagnostic laparoscopy identified inflammatory adhesions and a gastric ulcer perforation. Concurrent endoscopy precisely located the defect, which was effectively closed using an endoscopic overstitch device mounted on a therapeutic double-channel gastroscope. Closure involved three full-thickness inverted figure-of-eight sutures, verified by an intraoperative leak test and reinforced with an omental patch. Postoperative recovery was rapid and uncomplicated, progressing to a regular diet within 3 days. This innovative hybrid laparoscopic-endoscopic technique demonstrates efficacy, safety, and the benefits of minimally invasive surgery for pediatric gastrointestinal perforations, emphasizing its potential superiority over traditional methods.

## Introduction

Gastric ulcer perforation in children, although relatively uncommon, requires prompt intervention to prevent of severe complications [1, 2]. Nonsteroidal anti-inflammatory drugs (NSAIDs) are a recognized risk factor for the development of peptic-ulcer disease, which can progress to perforation [1]. Existing literature indicates that children undergoing long-term NSAID therapy are at an increased risk for developing asymptomatic gastric ulcers [2, 3].

The traditional management strategy for hollow viscus perforations has been surgical repair; however, there is a growing interest in exploring minimally invasive techniques that may offer advantages in terms of recovery and reduced morbidity [4]. This case report details a novel application of a laparoscopic-assisted endoscopic overstitch™ technique in a children, illustrating its feasibility and effectiveness as a potential alternative surgical approach.

## Case presentation

**Figure 1:**
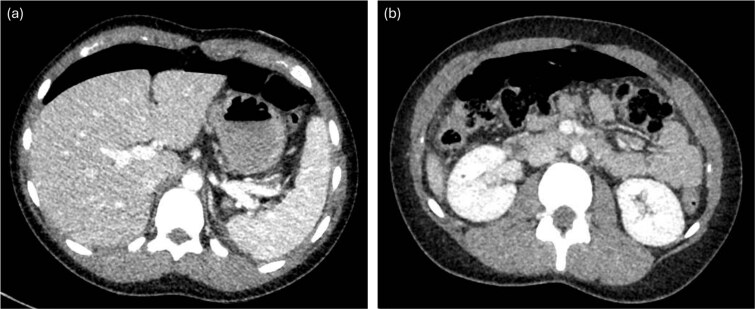
CT with intravenous contrast demonstrating large pneumoperitoneum.

A previously healthy 16-year-old female presented to the emergency department with acute severe abdominal pain, fever, nausea, and vomiting. She was tachycardic and febrile. Abdominal examination revealed diffuse tenderness and mild distension. An upright abdominal X-ray demonstrated pneumoperitoneum. A computed tomography (CT) scan of the abdomen and pelvis (Fig. 1) with intravenous contrast confirmed moderate free-air tracking from the liver to the bladder. Punctate air was also noted along the falciform ligament, overlying the right kidney, and near the left splenic-flexure, accompanied by mild perisplenic and perihepatic free fluid. Multiple air foci within the gastric body and antrum suggested gastric or duodenal ulcers. The patient had recently taken antibiotics for sinusitis and ibuprofen. She underwent immediate diagnostic laparoscopy.

### Technique

**Figure 2:**
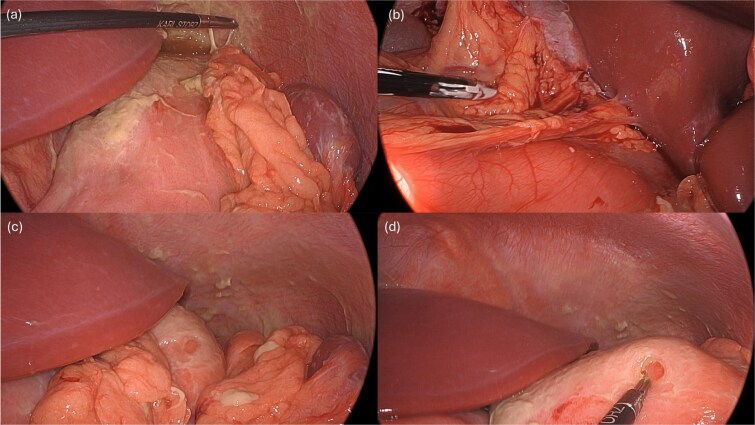
Intraoperative pictures demonstrating the sequelae of the hollow viscus perforation. The pictures in (a) and (b) demonstrate contamination and associated inflammatory adhesions in the left and right upper quadrant, respectively. The pictures in (c) and (d) demonstrate the antral perforation. The tip of the Maryland dissector was able to insert into the perforation (d) to assist with identification of the perforation endoscopically for closure.

Laparoscopy began with placement of a 5 mm umbilical trocar and two additional 5 mm trocars in the right and left abdomen. Turbid fluid was encountered and suctioned. In the upper-abdomen, significant inflammatory omental adhesions were found against the right upper quadrant and gallbladder. A complex fluid collection with inflammation was identified in the left upper quadrant (Fig. 2). These inflammatory adhesions were carefully dissected using a Cool-SealTM device to visualize the pylorus and duodenum; however, inflammation around the gallbladder prevented full release of these adhesions. Mobilization of the gastrocolic ligament revealed a normal posterior gastric wall. Because inflammatory exudates obscured the anterior stomach and the first and second part of the duodenum, making the perforation’s origin (duodenal or gastric) initially unclear, simultaneous intraoperative-endoscopy was requested for precise localization.

**Figure 3:**
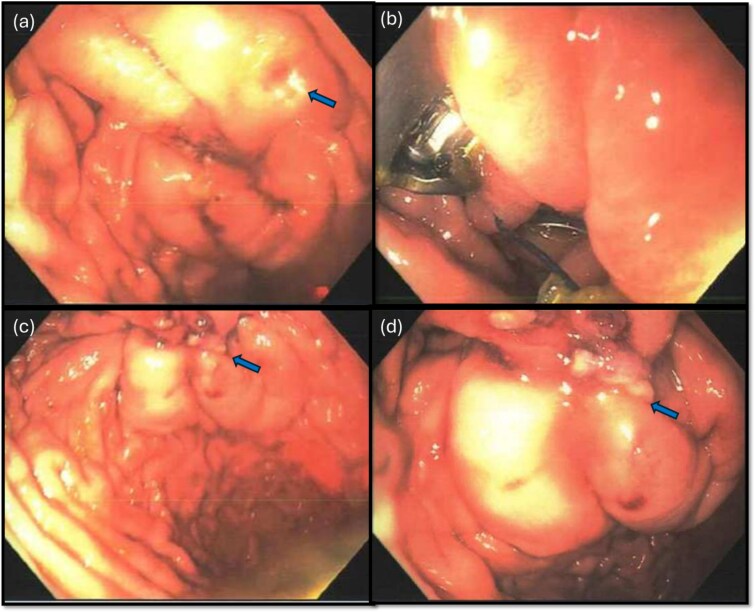
Endoscopic view of the perforation. The picture in (a) demonstrates the site of the perforation. The picture in (b) shows the use of the overstitch technique to perform the closure endoscopically. Pictures (c) and (d) demonstrate the area of perforation once the first and second layers of endoscopic closure are completed, respectively.

The gastroenterology team performed concurrent endoscopy using. With air insufflation and transillumination, a small pinpoint perforation was clearly identified on the anterior surface of the gastric-antrum (Figs 2 and 3).

To aid endoscopic visualization of the small perforation, a Maryland grasper was gently inserted into it (Fig. 2); the grasper’s tip, visualized endoscopically, confirmed the exact location. The remainder of the esophagus, stomach, and duodenum appeared normal. The Overstitch™ endoscopic-suturing device, mounted on a double-channel therapeutic endoscope and utilizing a tissue-helix for full-thickness bites, was used. Three full-thickness inverted figure-of-eight 2–0 Prolene sutures were placed at the perforation. Laparoscopic visualization confirmed full-thickness suture placement before the sutures were cinched, effectively closing the gastric wall defect (Fig. 3).

**Figure 4:**
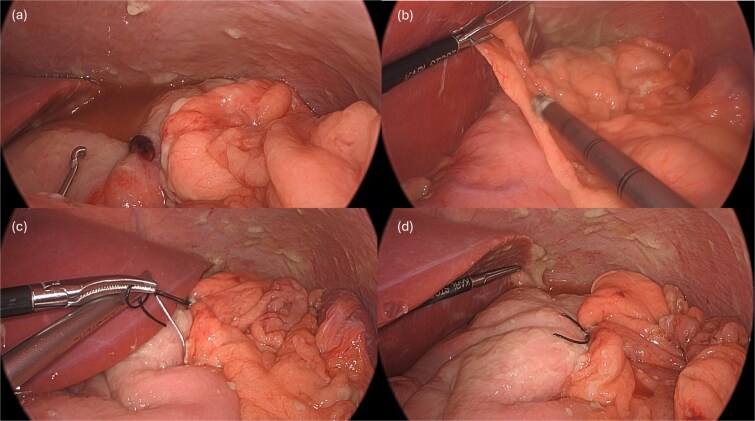
Following endoscopic closure, a leak test performed under water (a) did not demonstrate any bubbling after gastric insufflation with air. Photos (b–d) demonstrate performance of a graham patch with a redundant lip of omentum over the endoscopically closed perforation.

A leak test was then performed by submerging the repair site under saline and endoscopically insufflating the stomach; no bubbling was noted, indicating a successful seal. The repair was reinforced with an omental patch, secured laparoscopically with a 2–0 silk suture (Fig. 4). A single drain was left in the abdomen, remaining fluid was suctioned. Endoscopic biopsies of the stomach and duodenum were obtained, and a nasogastric tube was placed.

**Figure 5:**
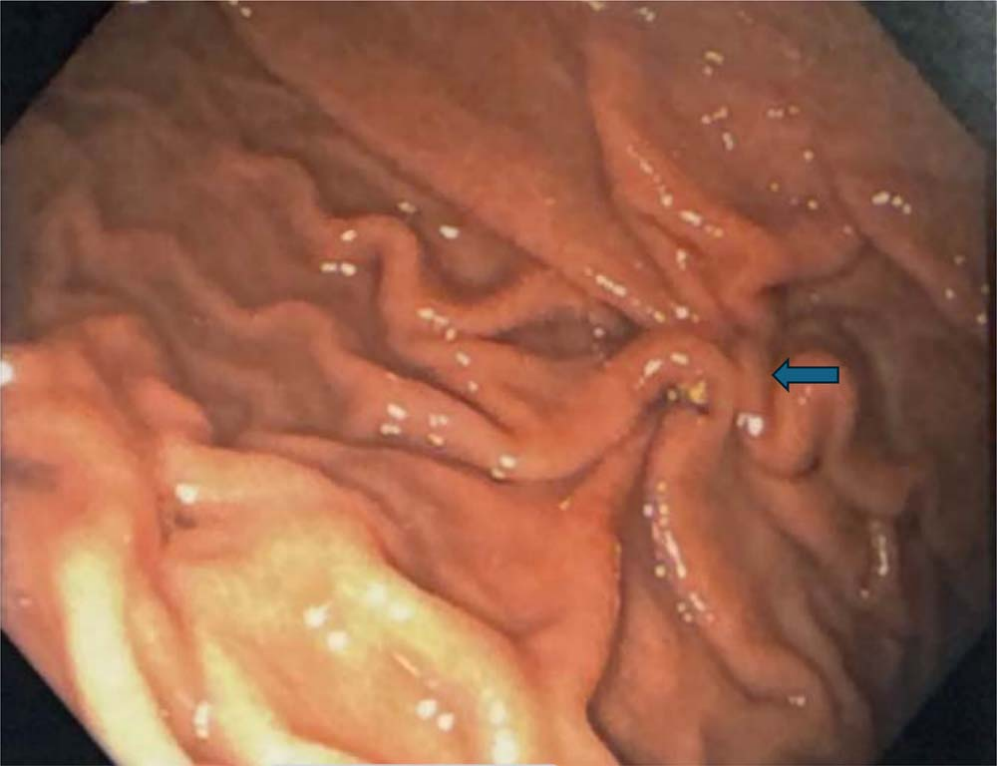
Follow-up endoscopy 3 months postoperatively demonstrating a well-healed suture line.

Postoperatively, the patient was admitted and received broad-spectrum antibiotics. An upper gastrointestinal series on postoperative day (POD) 1 demonstrated no leakage from the repair. The nasogastric tube was removed, and her diet was advanced from clear liquids to a regular diet by POD3. The peritoneal drain was removed on POD3, and the patient was discharged home. Final biopsy pathology revealed chronic inactive gastritis and tested negative for *Helicobacter pylori*. A follow-up endoscopy 3 months later showed a well-healed closure site (Fig. 5).

## Discussion

Overstitch™ was first studied in a porcine model to achieve successful closure of gastrocutaneous-fistulae [5]. This is a single-use, single-operator device that allows for placement of 2–0 polypropylene sutures [5, 6]. The device itself is mounted on distal end of the gastroscope and can be utilized in therapeutic double-channel gastroscopes and most recently in single channel gastroscopes. Overstitch™ devices consist of a needle-driver that transfer the needle to an anchor exchange catheter for needle exchange while suturing. It remains the only U.S. Food and Drug Administration-approved endoscopic-suturing device. It enables interrupted or continuous suture application and allows for full-thickness suturing for tissue approximation or plication in the gastrointestinal tract via the use of a tissue anchor, curved suturing arm, and a cinch [5, 6].

This report presents a novel hybrid approach to managing perforated gastric ulcers through the combined use of endoscopy and laparoscopy. Endoscopic suturing is a rapidly evolving minimally invasive technique that allows closure of larger defects compared to clip closure. Experience with the Overstitch™ endoscopic-suturing device suggests it may be superior to endoclips in the management of perforations because of its ability to achieve full-thickness suturing and create an airtight closure. Reviews indicate high closure rates for acute gastrointestinal defects, demonstrating that this technique is both safe and effective compared to traditional surgical interventions [7–9]. Notably, studies have reported a 100% success rate in some instances where the Overstitch™ was employed to manage postoperative leaks, underscoring its utility in scenarios where conventional methods may fall short [7–9]. The literature suggests that endoscopic-suturing techniques provide a minimally invasive alternative, potentially reducing the need for more invasive surgical procedures [5].

The use of advanced endoscopic techniques to manage complications in children, including suturing and defect closure, are still in their infancy [10–12]. While endoscopic management of upper gastrointestinal perforations in children has been reported with gastroscopic closure using clips [12], the direct application of the Overstitch™ technique in children appears to be limited. Many cases in children continue to depend on traditional surgical approaches, largely due to the unique complexities and anatomical considerations inherent to younger patients. This report represents the first documented use of this laparoscopic-endoscopic hybrid technique for managing perforations in children, highlighting the potential for broader application of minimally invasive approaches in addressing complex gastrointestinal conditions in this population.

## References

[1] Coxib, traditional NTC, Bhala N, Emberson J, et al. Vascular and upper gastrointestinal effects of non-steroidal anti-inflammatory drugs: meta-analyses of individual participant data from randomised trials. Lancet. 2013;382:769–79. 23726390 10.1016/S0140-6736(13)60900-9 PMC3778977

[2] Wong BP, Chao NS, Leung MW, et al. Complications of peptic ulcer disease in children and adolescents: minimally invasive treatments offer feasible surgical options. J Pediatr Surg 2006;41:2073–5. 10.1016/j.jpedsurg.2006.08.009 17161209

[3] Hollingworth P . The use of non-steroidal anti-inflammatory drugs in paediatric rheumatic diseases. Br J Rheumatol 1993;32:73–7. 10.1093/rheumatology/32.1.73 8422565

[4] Edwards MJ, Kollenberg SJ, Brandt ML, et al. Surgery for peptic ulcer disease in children in the post-histamine2-blocker era. J Pediatr Surg 2005;40:850–4. 10.1016/j.jpedsurg.2005.01.056 15937829

[5] Ge PS, Thompson CC. The use of the overstitch to close perforations and fistulas. Gastrointest Endosc Clin N Am 2020;30:147–61. 10.1016/j.giec.2019.08.010 31739961 PMC6885379

[6] Kantsevoy SV . The development of the overstitch system and its potentials. Gastrointest Endosc Clin N Am 2020;30:107–14. 10.1016/j.giec.2019.08.004 31739957

[7] Hustak R, Vackova Z, Krajciova J, et al. Endoscopic clips versus overstitch suturing system device for mucosotomy closure after peroral endoscopic pyloromyotomy (G-POEM): a prospective single-center study. Surg Endosc 2022;36:9254–61. 10.1007/s00464-022-09417-1 35851820

[8] Jinushi R, Tashima T, Fujita A, et al. Conventional clips vs over-the-scope clips for mucosal defects closure after duodenal endoscopic submucosal dissection. Gastro Hep Adv 2023;2:1034–9. 10.1016/j.gastha.2023.07.004 39131547 PMC11308691

[9] Kolb JM, Hammad H. The use of the overstitch to close endoscopic resection defects. Gastrointest Endosc Clin N Am 2020;30:163–71. 10.1016/j.giec.2019.08.006 31739962 PMC7202237

[10] Alqahtani A, Elahmedi M, Alqahtani YA, et al. Endoscopic sleeve gastroplasty in 109 consecutive children and adolescents with obesity: two-year outcomes of a new modality. Am J Gastroenterol 2019;114:1857–62. 10.14309/ajg.0000000000000440 31658128

[11] Lerner DG, Mencin A, Novak I, et al. Advances in pediatric diagnostic endoscopy: a state-of-the-art review. JPGN Rep 2022;3:e224. 10.1097/PG9.0000000000000224 37168622 PMC10158303

[12] Sharma S, Barakat M, Urs A, et al. Applicability, efficacy, and safety of over-the-scope clips in children. Gastrointest Endosc 2022;95:489–99. 10.1016/j.gie.2021.10.011 34662583

